# Spatially resolved imaging of human macular capillaries using adaptive optics-enhanced optical coherence tomography angiography

**DOI:** 10.1038/s41598-024-65534-y

**Published:** 2024-07-05

**Authors:** S. Bonnin, K. Gocho, N. Norberg, E. Gofas, R. Lejoyeux, C. Chaumette, K. Grieve, A. Couturier, M. Paques

**Affiliations:** 1 Paris Eye Imaging Group, Clinical Investigation Center Vision 1423, Quinze-Vingts Hospital, INSERM-DHOS, Sorbonne University, 28 Rue de Charenton, 75012 Paris, France; 2 Rothschild Foundation Hospital, 25-29 Rue Manin, 75019 Paris, France; 3 Institut de La Vision, 17 Rue Moreau, 75012 Paris, France; 4 Université Paris Cité, Ophthalmology Department, AP-HP, Lariboisière Hospital-Assistance Publique-Hôpitaux de Paris, Paris, France

**Keywords:** Retina, Microcirculation, Optical coherence tomography angiography, Macula, Fovea, Optical physics, Anatomy, Medical research

## Abstract

Documenting the organization of the retinal capillaries is of importance to understand the visual consequences of vascular diseases which may differentially affect the microvascular layers. Here we detailed the spatial organization of the macular capillaries in ten healthy human subjects using a prototypic adaptive optics-enhanced optical coherence tomography angiography (AO-OCTA) system. Within the central 6° × 6°, the radial peripapillary capillaries and the superficial, intermediate and deep vascular plexuses (SVP, IVP and DVP, respectively) were consistently resolved. In 8 out of the 10 eyes, the capillary segments composing the perifoveal arcade (PFA) were perfused only by the SVP, while drainage of the PFA showed more variability, comprising a case in which the PFA was drained by the DVP. Around the center, a distinct central avascular zone could be documented for each layer in 7 of the 10 cases; in three eyes, the IVP and SVP merged tangentially around the center. In all eyes, the foveal avascular zone was larger in the DVP than in the SVP and IVP. In one eye with incomplete separation of the inner foveal layers, there was continuity of both the SVP and the IVP; a central avascular zone was only present in the DVP. The diversity of perfusion and drainage patterns supported a connectivity scheme combining parallel and serial organizations, the latter being the most commonly observed in perifoveal vessels. Our results thus help to further characterize the diversity of organization patterns of the macular capillaries and to robustly analyze the IVP, which will help to characterize early stages of microvascular diseases.

## Introduction

An optimal angioarchitecture is critical for metabolic support of neural activity. In the mammalian holangiotic retina, the blood flow is distributed through a multilayered capillary meshwork^1–4^. In primates, four microvascular plexi have been described: the radial peripapillary capillaries (RPCs), characterized by long capillary segments parallel to the direction of ganglion cell axons; the superficial vascular plexus (SVP), which is the main source of arterial flow, arranged in a lattice pattern; and, bracketing the inner nuclear layer (INL), are the intermediate (IVP) and deep (DVP) vascular plexuses^2^.

A comprehensive knowledge of the retinal angioarchitecture has to take into account the fact that its organization varies with retinal locations in particular its variations with retinal thickness^1,3^. The latter is however not the sole determinant of the vascular density; indeed, the capillary density is higher where the retinal nerve fiber layer (RNFL) is the thickest, that is, around the optic disc, reflecting higher metabolic needs of this layer^1^. Furthermore, a specialized region called the macula supports the highest visual acuity and has also a specific organization. The macula has a thickened rim, the clivus, which has a high concentration of ganglion cells, and is centered by the fovea, the thinnest retinal zone, which contains the highest density of photoreceptors. The spatial organization of the vascular network of the macula was first documented in non-human primates in a seminal histology study^1^. In the center of the macula is the foveal avascular zone (FAZ) which is bordered by arcuate capillary segments forming the perifoveal arcade (PFA)^1,4^. The absence of vessels in the foveal center is thought to improve central vision by minimizing lightscatter, although subjects with vessels in the fovea may have normal vision^5,6^. The diameter of the PFA closely matches the width and depth of the foveal pit^7–9^. The PFA has a clinical significance disproportionate to its flow. The PFA is indeed the closest source of oxygen for the synapses of foveal cones^10,11^. Thus, minute capillary defects in the PFA such as those seen in diabetic retinopathy may compromise vision. Furthermore, diabetic retinopathy may differentially affect the microvascular layers^12^*,* highlighting the importance of accurate resolution of each layer. Therefore, understanding the distribution of the blood flow in the PFA, is of paramount clinical interest to understand visual consequences of microvascular diseases in humans. Nevertheless, in sharp contrast with its clinical importance, to our knowledge no comprehensive description of macular capillaries has been reported.

The distribution of flow determines the efficiency of metabolic supply and the capability to respond to pathological situations. Hence it is important to determine the extent to which the different layers are connected. The connection pattern may also influence the length of the capillary pathway and thus the delivery of metabolites. A serial organization can be described as the passage of flow through all layers, while a parallel organization would mean that each layer has distinct inflow and outflow pathways. The relative importance of one scheme versus the other, which may vary among species^13,14^ and among retinal zones, is still debated. Arguments for a preferential serial or parallel organization of the human retina have been reported. Histology supports both serial and parallel connectivity patterns of the macula^15^; yet, in vivo data diverge somewhat from histology^2^. Histology of macular vessels, in particular in the fovea, is however hindered by several factors, among which fixation artifacts that may deform fovea hence rendering difficult the appreciation of microvascular layering. Furthermore, it is rather cumbersome and good quality samples are scarce. Given the large phenotypic spectrum of the fovea^8^, histology is conceivably not the most efficient strategy to document the diversity of its microvascular organization. On the contrary, optical coherence tomography angiography (OCTA) gives access to the capillary bed of the living human retina in an unbiased, noninvasive manner^16,17^. Using OCTA in healthy eyes, it has been postulated that each of the three capillary plexuses has its own feeding arteriolar supply and draining venules, suggesting a preferential parallel connectivity pattern^18^. On the opposite, based on the preferential development of post-vein occlusions collaterals in the DVP, it has been postulated that the circulation pattern has predominantly a serial vascular arrangement^19^.

Currently commercially available OCTA technologies have notable limitations because the diameter of retinal capillaries is close to the xyz resolution of conventional OCTA, so many capillaries are displayed with discontinuities, and the distinction of microvascular layers, especially the IVP, is often plagued by overlap of adjacent layers. The pathophysiological importance of the IVP has been suggested in previous histology and clinical papers^20,21^. Optical aberrations may be one of the causes of such limitations of resolution of OCTA. Salas et al. first demonstrated the interest of using adaptive optics (AO) combined with OCTA to better segment the different capillary plexuses in the retina^22^. Here, thanks to a novel AO-corrected OCTA system^23^, we explored the organization of microvessels in the normal human fovea at the single capillary level.

## Results

**Figure 1:**
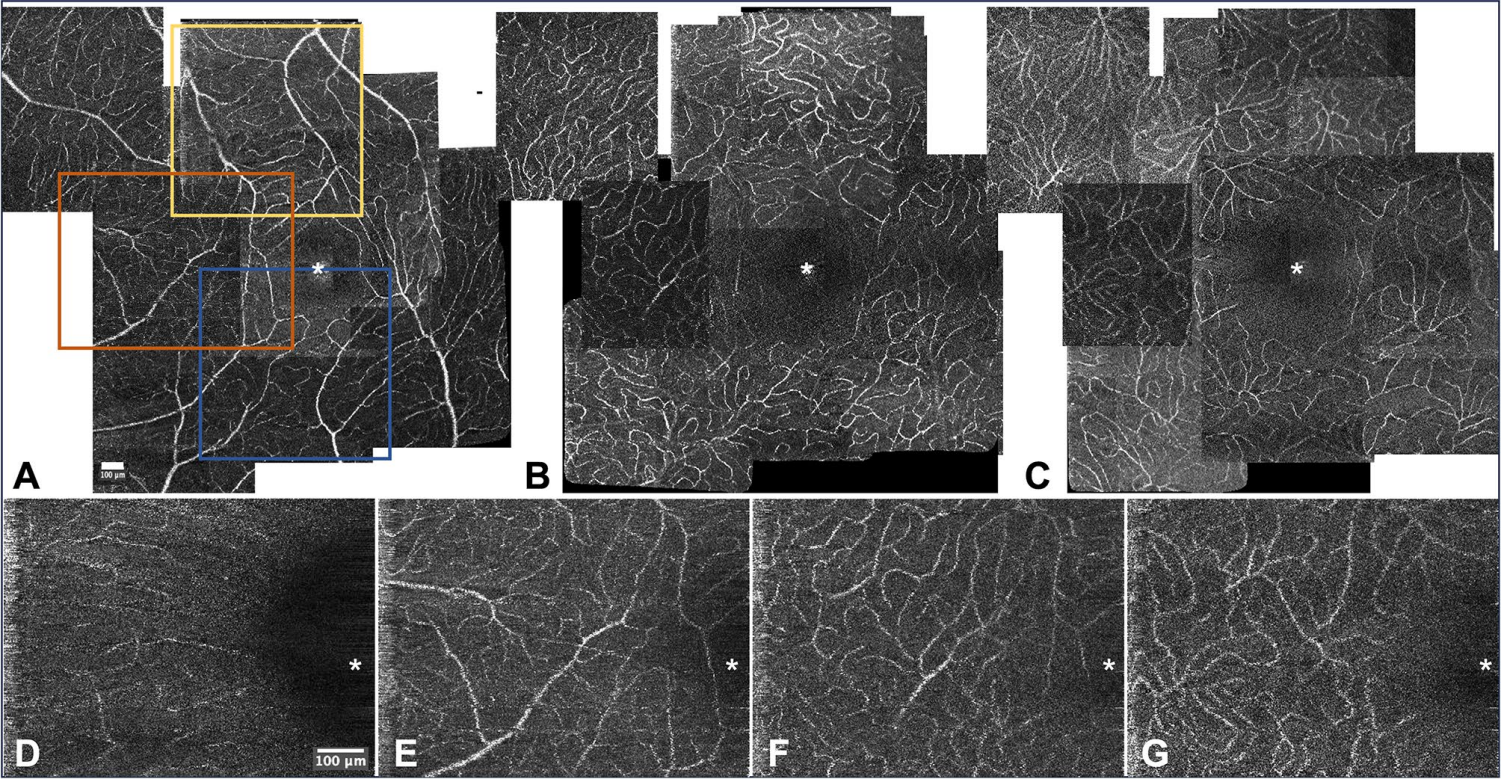
Adaptive optics optical coherence tomography angiography (AO-OCTA) imaging of the microvascular plexuses of subject 1. A to C: Montages of the superficial (SVP) (**A**), intermediate (IVP) (**B**) and deep vascular plexus (DVP) (**C**). The fovea is marked with an asterisk in the SVP. D to G: Magnification of the orange rectangle from A showing the radial peripapillary capillaries (**D**), the SVP (**E**), the IVP (**F**), and the DVP (**G**). The yellow and the blue rectangles correspond to Supplementary Video 1 and Supplementary Video 2, respectively. All scale bars represent 100 microns.

We obtained detailed, spatially resolved mapping of the capillaries in the macula of 10 normal eyes (Table 1). AO-OCTA allowed distinguishing each layer with minimal overlap, enabling to unambiguously assign a given capillary to a given layer. In particular, we were able to isolate the IVP. Figure 1 and supplementary videos 1 and 2 show montages of AO-OCTA images of subject 1, in which the RPCs, SVP, IVP and DVP are clearly distinguished. RPCs were identified in areas with the thickest RNFL, i.e., in the nasal half of the macula. Compared to the other layers, the contrast of the RPCs was lower, possibly due to the background noise from the RNFL. The SVP was composed of second-order arterioles and venules connected by horizontal capillaries that were generally arcuate, concentric to the FAZ. Some capillaries of the SVP were seen to bifurcate toward the IVP. The IVP and DVP were a meshwork of tortuous capillaries organized into clusters of converging capillaries. The confluence of tortuous capillaries and post-capillary venules toward vein defined what we called "venous confluences" also referred to as “vortexes” in the literature^16^. These venous confluences were identified in all layers except in the RPCs. In the IVP and DVP, these confluences were more clearly visible, and were centered by vertical (connecting) venules (Suppl. Figure 1). These venous confluences were less numerous in the IVP than those in the DVP. The most distinctive feature of the DVP compared to the other layers was that venous confluences were interconnected to each other, hence the DVP appeared as a continuous mosaic of venous confluences.

**Table 1:** Characteristics of the study subjects (F: female, M: male, LE: left eye, RE: right eye, NA: non available).

	Age (years)	Gender	Studied eye	Spherical Equivalent (diopters)	Axial Length (mm)
Subject 1	38	F	LE	+ 0.5	23.93
Subject 2	59	M	RE	+ 0.5	24.68
Subject 3	36	F	RE	− 4.25	23.82
Subject 4	29	F	RE	− 1.25	23.44
Subject 5	34	M	RE	+ 0.75	22.75
Subject 6	35	M	LE	0	23.84
Subject 7	31	F	RE	− 2	23.44
Subject 8	33	M	LE	+ 6,5	22.13
Subject 9	28	M	LE	0	24.59
Subject 10	35	F	RE	− 4	NA

**Figure 2:**
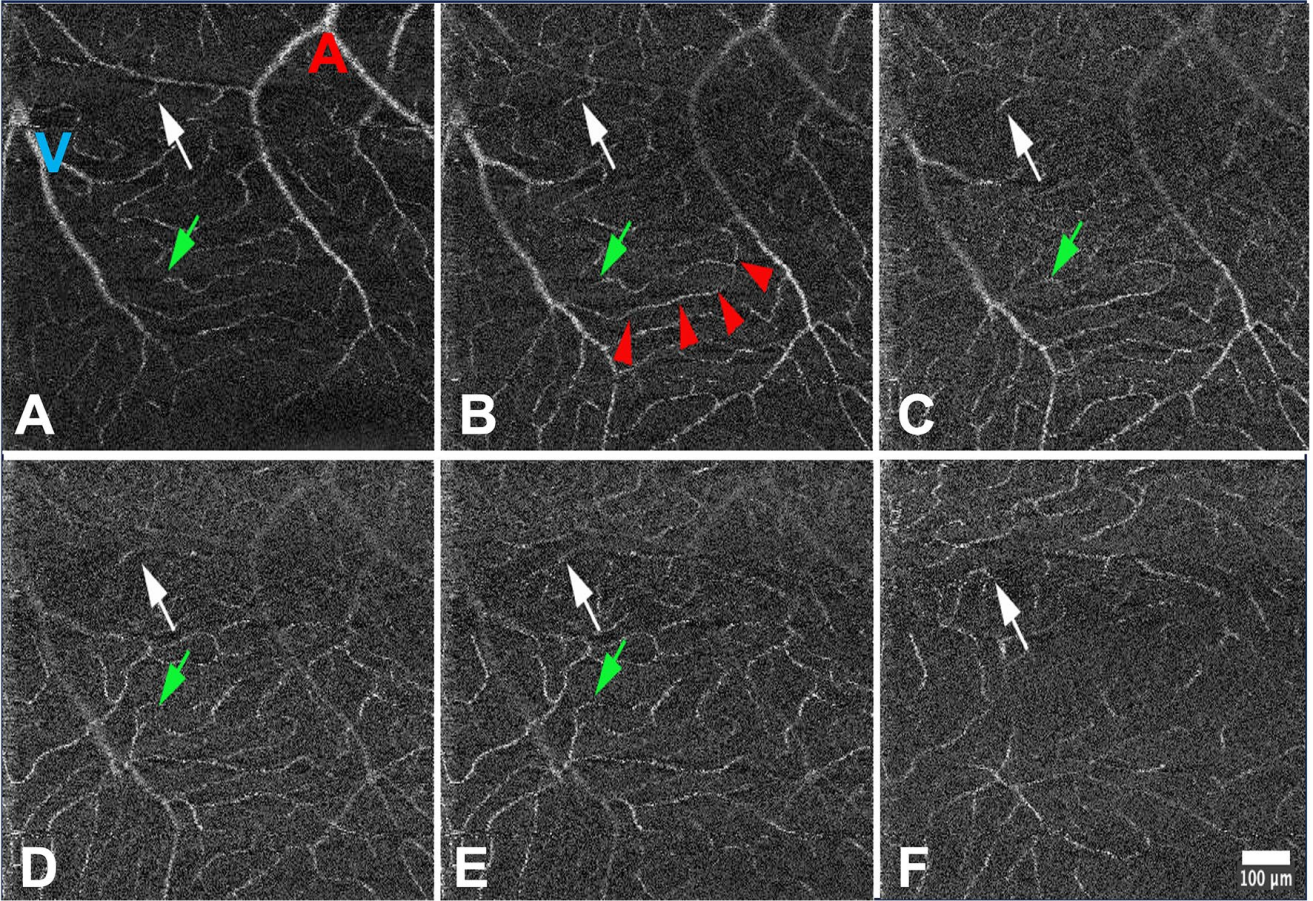
Illustration of different connectivity patterns of an arteriole in the SVP from subject 1 (magnification of the yellow rectangle in Fig. 1, see also supplementary video 1). A to F: successive AO-OCTA slabs from the SVP (**A**) to the IVP (**F**). The white arrows follow a vessel emerging from an arteriole that perfuses the IVP without branching into the SVP. The green arrows follow a capillary in the SVP that branches into the IVP. The red arrowheads follow a capillary that drains in the SVP (A: arteriole, V: venule).

**Figure 3:**
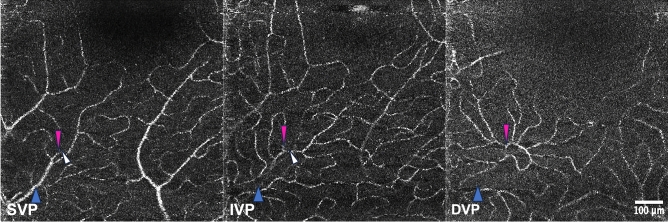
Illustration of venous connectivity patterns in subject 1 (blue rectangle in Fig. 1). The presumed location of the transverse (vertical) vein is indicated by the blue arrows. The white arrows show the emergence of a vein from the IVP to the SVP. The pink arrows show an example of a vein emerging from the DVP to the SVP without connecting to the IVP. The presence of separate, superimposed venous confluences in the three layers suggests that the SVP and IVP each have their own drainage pathway (See also Supplementary Video 2).

Patterns of connections between layers were analyzed by tracking individual capillaries. Figure 2 and Supplementary Video 1 show different types of connections emerging from the SVP. There were arterioles of the SVP that diverged downward to the IVP, i.e. without perfusing capillaries in the SVP. We found no direct connections of the SVP arteries to the DVP. Capillaries of the SVP either bifurcated to the IVP or drained into post-capillary venules of the SVP. In the IVP, connections with upstream and downstream vessels were identified, as well as direct venous drainage, i.e., without connection to the DVP (Fig. 3 and Suppl. Figure 1). Venous confluences from different layers were in some cases superimposed, suggesting the presence of a vertical venule emerging from the DVP connecting these complexes. An example of the superimposition of venous confluences is shown in Fig. 3 and in Supplementary Video 2. Some transverse venules from the DVP traversed the IVP without connecting to it.

**Figure 4:**
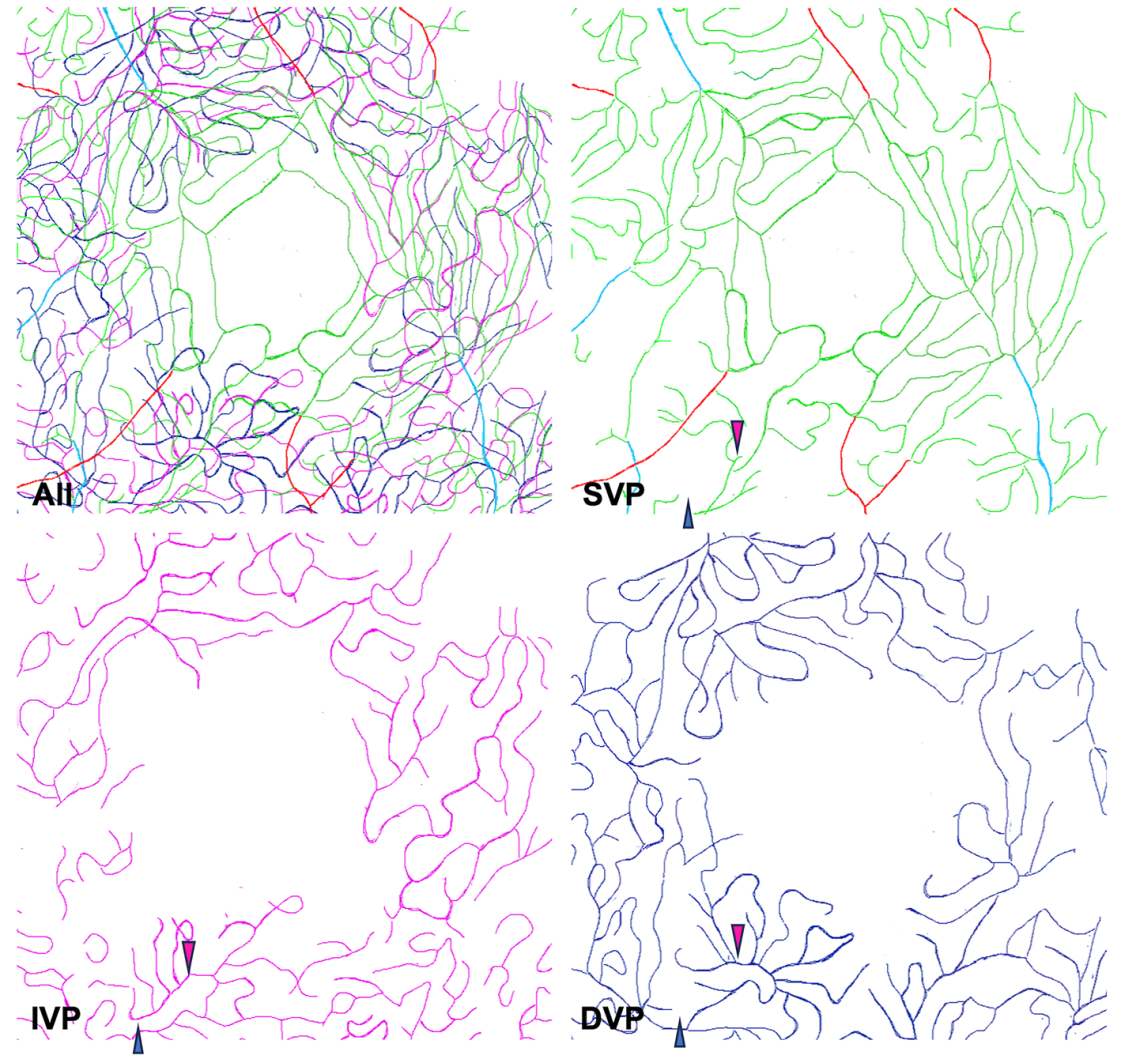
Tracings of the perifoveal area of subject 5 splitted by capillary layers. The perifoveal area is supplied by the SVP and drained by the DVP. Note the precapillary arteriole contributing to the PFA. The diameter of the circles fitting the FAZ were 260 µm, 290 µm and 370 µm in the SVP, IVP and DVP, respectively.

**Figure 5:**
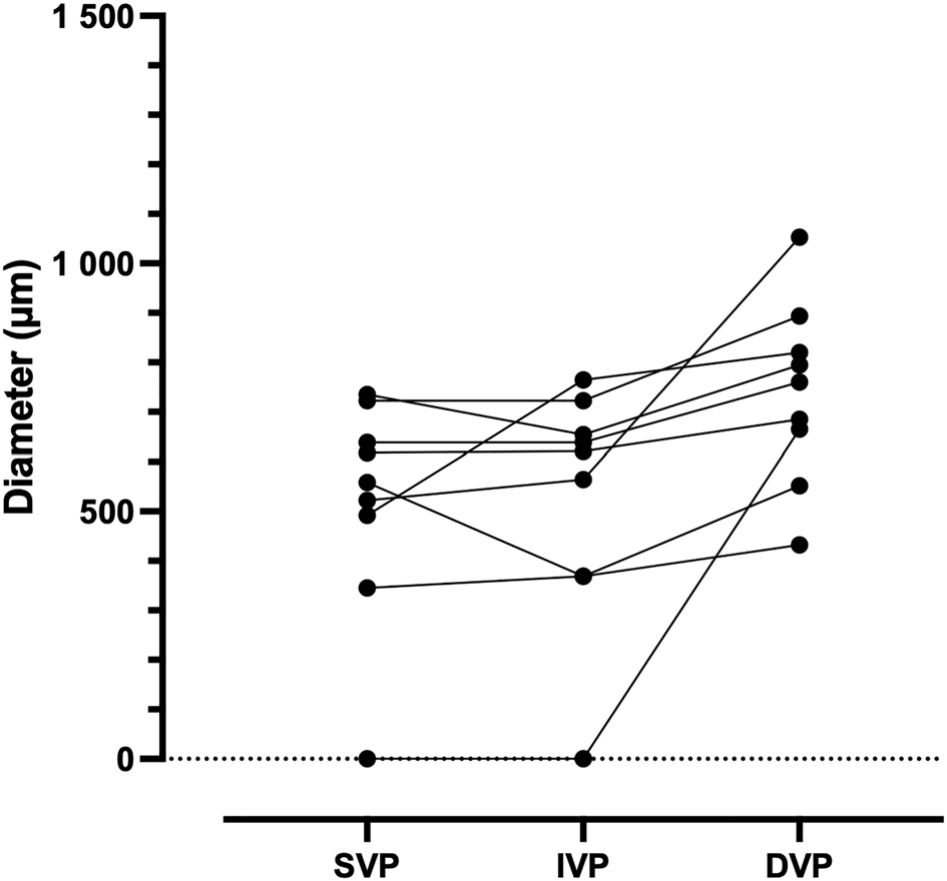
Comparison of the central capillary-free zones of the SVP, IVP and DVP for each patient (y axis: diameter of the largest fitting circle in µm). Each line represents one patient.

The PFA was formed of arcuate, interconnected capillary segments mostly perfused by the SVP, while in the IVP and DVP the inner limits of capillaries were associating loops and cross-sectioned capillaries (Fig. 4). The PFA was perfused solely by the SVP in 8 out of 10 eyes. Drainage of the PFA associated equally the SVP and IVP, with contribution from the DVP in one case. In subject 5, a precapillary arteriole of the SVP contributed to the PFA (Suppl. Figure 2 and Suppl. Video 3). In subject 9, the PFA was perfused by the IVP and drained by the DVP (Suppl. Video 4). The central interruption of capillaries occupied different surfaces areas in the SVP, IVP and DVP. Average values (± SD) for the diameter of the largest fitting circle of the central capillary-free zone in the SVP, IVP and DVP were 515 µm (± 228), 522 µm (± 240) and 740 µm (± 184), respectively (p = 0.027). Comparison of their respective size (Fig. 5) showed that capillary-free zones were larger in the DVP.

**Figure 6:**
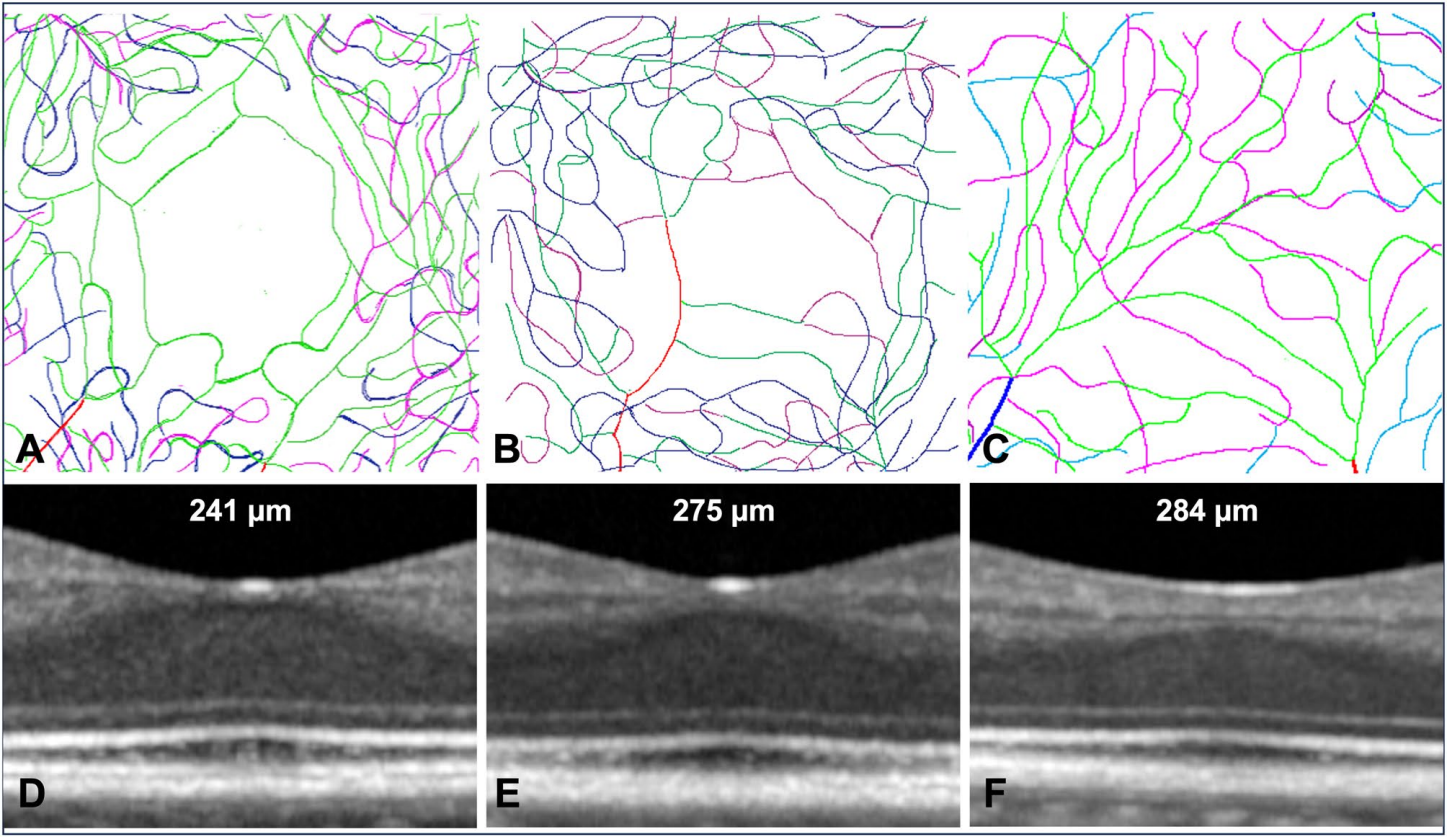
Correlation between neuronal and vascular structures. Tracings of subjects 1 (**A**), 5 (**B**) and 8 (**C**) and corresponding B-scans (**D**, **E** and **F**). The capillaries of the SVP are traced in green, the capillaries of the IVP are traced in magenta and the capillaries of the DVP are traced in blue. The central foveal thickness is indicated on each B-scan (**D**, **E** and **F**). Note that the slight difference in foveal structure between subjects 5 and 8 contrasts with the profound changes in foveal capillary coverage.

**Figure 7:**
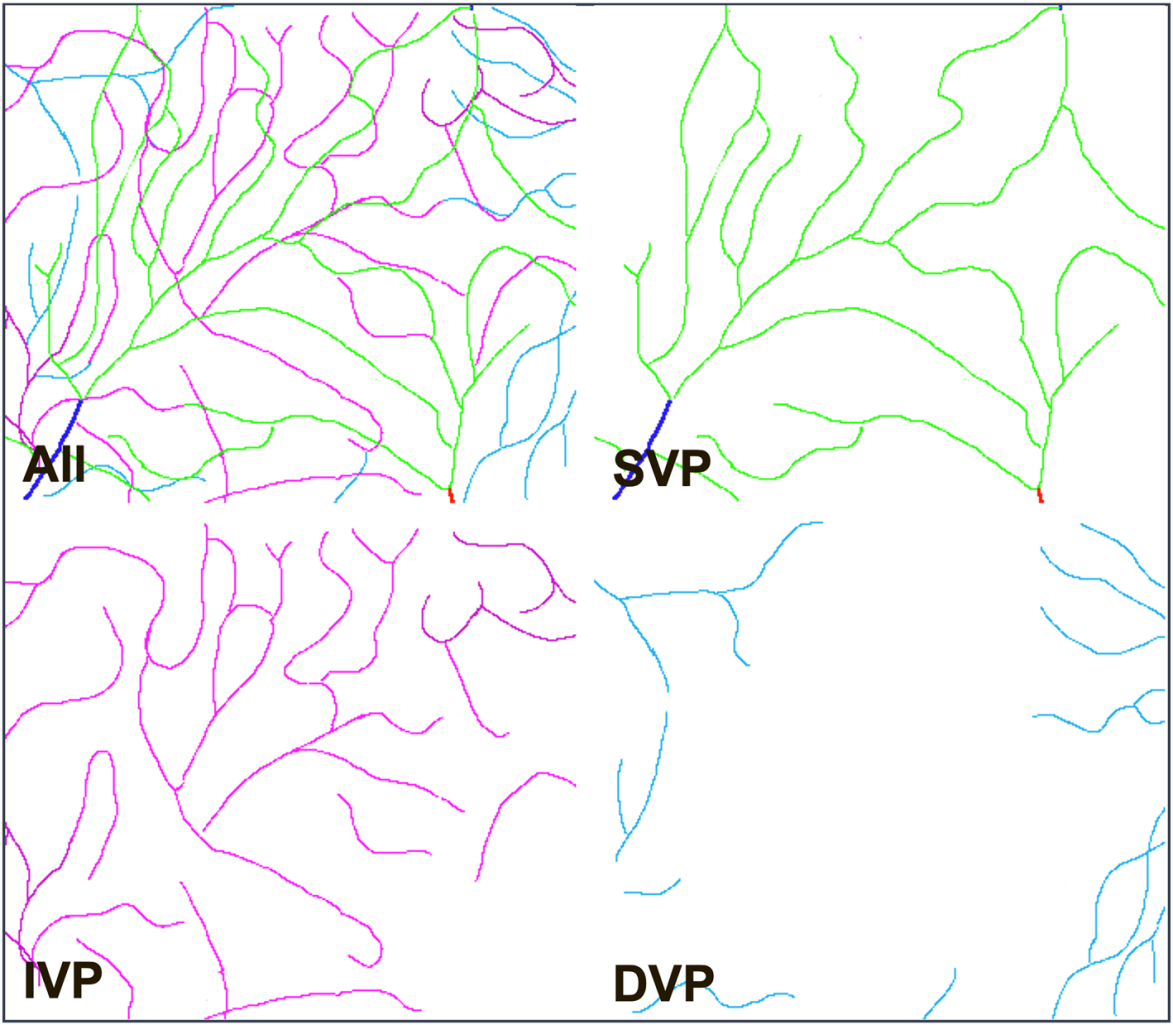
Tracings of perifoveal vessels in subject 8 splitted by capillary layers. The central avascular zone is only visible in the DVP (See also supplementary video 5). Note that the SVP over the center forms a venous confluence.

Finally, we documented the relationship of microvascular and neuronal organizations. Figure 6 illustrates the correspondence between neuronal and vascular structure in the foveal center in three subjects with increasing foveal thickness. In subject 8, who had no separation of the inner layers in the foveal center, a central capillary interruption was detected only at the level of the DVP (Fig. 7). The thickness of central inner retinal neurons in subject 7 was 22 µm and that of subject 8 was 38 µm; only in the latter was the FAZ absent, with the SVP and the IVP being separated by 21 µm. Of note, the width of the central defect of the DVP was within the range of the other subjects, suggesting that the size of the central defects is differentially regulated.

## Discussion

In this study, a detailed in vivo mapping of the macular capillaries of normal eyes was performed. Our prototypic AOOCTA was able to unambiguously isolate the IVP from adjacent layers and to document a diversity of connection patterns of perifoveal vessels. The main findings were that in most eyes, the SVP was the unique source of the PFA while drainage patterns showed more variability, and distinct width of central interruptions of capillaries within layers were identified.

The organization of the perifoveal capillaries, especially the inflow and outflow patterns of the PFA, is still incompletely deciphered. To our knowledge, there has been no model of the PFA connections of the human macula. The fact that there is a central interruption of capillary coverage in the center implies that all three layers are interrupted. The existence of differing values for the central capillary-free zone in the inner and outer vascular layers has previously been documented using conventional OCT^9,24,25^. This is in particular well documented in fovea plana. The question that emerges is whether there is fusion of the microvascular layers or wether there are distinct interruptions for each layer. Indeed, two arrangements may be postulated: either the layers converge, in parallel with the progressive thinning of inner layers as they approach the fovea, or each capillary around the fovea terminates by a change in direction toward adjacent layers through transverse capillaries. In our patients, a FAZ could be clearly documented in 7 eyes in the 3 plexuses; in 3 eyes we were unable to define two different FAZ for the SVP and the IVP, while a FAZ in the DVP was more clearly evident. The diameter of these avascular zones was larger in the DVP than in the SVP except in one case.

Determining the connectivity pattern of the microvascular layers is of importance to understand retinal physiology and pathology. As there is evidence of both patterns in the human macula, the overall connectivity is necessarily hybrid; however, a predominance of one arrangement over the other cannot be excluded. This would require quantitative analysis of the connections, which remains to be performed. Of interest is to note that the PFA is in most cases perfused by the PFA and drained by the IVP, hence having a serial connectivity scheme. In light of our findings, we analyzed the scheme of primate macular capillaries proposed by Snodderly^1^. As in humans, most of the drainage of the PFA in that scheme occurred through the IVP, suggesting that there is serial organization of the SVP and IVP in perifoveal vessels in this specie. If future works confirm that there is no arterial perfusion of the DVP, then it would appear that the perfusion scheme is preferentially serial. On the other hand, the drainage pattern appears to be preferentially parallel as shown by the presence of venous confluences in each layer.

Capturing fine details of the perifoveal vasculature increases our capability to analyze neurovascular relationship at the microscopic level. During development, formation of the foveal pit begins at 25 fetal weeks and excavation is complete 15–45 months after birth^26^. Disruption of this developmental process results in fovea plana (also called foveal hypoplasia) which can be associated with conditions such as albinism, *PAX6* mutations or can occur in isolation^22,23^. It is known that in patients with a reduced or absent foveal pit, the FAZ is smaller or even absent which affects more commonly the SVP^27,28^. Here, in two subjects having continuity of the inner retina over the fovea, we observed that there was in one case presence of a small FAZ (and hence interruption of the three layers), and in the other continuity of the SVP and IVP while the DVP was interrupted. Hence, a relatively small increase in central inner layer thickness (from 22 to 38 µm) was associated with a marked change in the organization of the foveal perfusion with disappearance of central avascular zones of the SVP and IVP. This illustrates the interest of AOOCTA to document neurovascular relationship at a very fine level.

Such details of capillary organization may also be of interest to better interpret the data from conventional OCT. It is known that vascular diseases may affect differentially each layer. The poor discrimination of the IVP in many OCTA systems may lead to an underestimation of the surface area of capillary nonperfusion. This may be the case for instance when there are superimposition of venous confluences, because the occlusion of one of them in a single layer may be unnoticed by conventional OCTA.

In summary, the disposition and connectivity of macular capillaries was analyzed using AOOCTA in ten healthy human subjects. Documenting the various patterns of macular circulation is essential for understanding the visual consequences of microvascular diseases. We found that in most eyes the perfusion of the PFA involved only the SVP, while the drainage pattern involved all layers to a variable extent. Around the fovea, the organization of capillaries around the FAZ was detailed, providing evidence that each microvascular layer forms a distinct avascular zone of varying size. The presence of perfusion and drainage vessels bypassing the DVP indicates the existence of a hybrid distribution pattern combining serial and parallel organizations. Further exploration of the spectrum of foveal morphologies will be of interest to better understand developmental and pathological neurovascular interactions. In particular, a better knowledge of the early steps of diabetic retinopathy are expected.

## Methods

This study was conducted in accordance with the tenets of the Declaration of Helsinki. Written informed consent was obtained from all participants following an explanation of experimental procedures and risks, both verbally and in writing. All experiments were approved by the appropriate ethics review boards in France (Comité de Protection des Personnes and Agence Nationale de la Santé et du Médicament (registered in clinicaltrials.gov NCT04129021; IDRCB number: 2019-A00942-55). Five were 5 women and 5 men (4 left eyes and 6 right eyes) were included. The mean age was 37 years (standard deviation [SD]: 8.7; range: 28–59) (Table 1)*.* None of the subjects had significant medical or ocular comorbidities.

Standard Optical Coherence Tomography (OCT) imaging was performed using Spectral-Domain OCT combined with a scanning laser ophthalmoscope (Spectralis OCT-SLO; Heidelberg Engineering, Heidelberg, Germany). The foveal center was identified as the deepest point of the foveal pit, containing a central light reflex, and the central foveal thickness was manually measured at this point. AOOCTA volume data were collected using a previously described clinical prototype^23^. This device provided SLO, OCT and OCTA. The OCT/OCTA modalities were based on a swept-source operating at a frequency of 200 kHz, with a central wavelength of 1050 nm and a bandwidth of 110 nm. The imaging system was equipped with a magnification switch, allowing for the selection of either a low or high optical magnification setting. The low magnification setting corresponded to a field size of 40° × 30° while the high magnification setting corresponded to a field size of 4° × 3° °. When images were acquired under high magnification, wavefront aberrations were compensated throughout the procedure using an integrated closed-loop AO system operating at 10 Hz. The lateral optical resolution of AOOCT/AOCTA was approximately 4.1 µm. During image acquisitions, the position of the system with respect to the subject's eye was continuously adjusted in three dimensions by motorized translation stages driven by a pupil tracker. Eye motion was also compensated in OCT/OCTA scans by an SLO-based retinal tracker operating at 400 Hz. Further technical details can be found in a previous article^23^. In the majority of cases, the AOOCTA volume data were obtained by scanning 300 lines in each region of interest, with four repeated B-scans on each line. Each B-scan included 400 A-scans. The voxel size was 2.7 µm along the axial dimension and 3 µm × 3 µm along the two other (lateral) dimensions. In a few cases, a slightly different scan pattern was employed, comprising 400 lines instead of 300 and a lateral voxel size of 2.25 µm instead of 3 µm.

As a rule, post-processing of the images was performed as follows: AO-OCTA stacks with distortion artifacts were deleted. Since the *en face* section was not always congruent with the plane of the vascular plexus, the same microvessel layer could appear on different images. Therefore, by scrolling the stack in the z-axis, microvessel layers were identified on each image and manually cropped using Fiji (available in the public domain at rsb.inf.nih.gov/ij; National Institutes of Health, Bethesda, MD). The cropped images were then collected and a 2D maximum intensity projection was computed using the z-projection function. This process was repeated for each layer as necessary. Montages of the computed images were performed using i2k Align Retina (DualAlign, LLC, Clifton Park, NY), and additional manual corrections were performed using Adobe Photoshop 7.0 (Adobe Corporation, Mountain View, CA) when necessary. AO-OCTA images were reviewed by three of us (SB, KG, MP). Ambiguities in image interpretation, particularly in the assignment of a particular capillary to a particular layer, led to re-examination of the AO-OCTA sequence until agreement was reached. Capillaries were then manually traced and color-coded using Adobe Photoshop 7.0. The size of the central avascular zone in each layer was defined as the diameter of the largest fitting circle. Arterioles and venules were identified by comparison with large field images.

### Supplementary Information

Supplementary Information 1.

Supplementary Information 2.

Supplementary Information 3.

Supplementary Information 4.

Supplementary Information 5.

Supplementary Information 6.

## Data Availability

The data generated and/or analyzed in this study are available from the corresponding author on reasonable request provided that a data processing agreement is completed and fulfilled.
