# Supplementary figures and images for: Spatially resolved imaging of human macular capillaries using adaptive optics-enhanced optical coherence tomography angiography
Source: Sci Rep. 2024 Jul 5;14:15540. doi: 10.1038/s41598-024-65534-y (PMC11226425; doi:10.1038/s41598-024-65534-y)

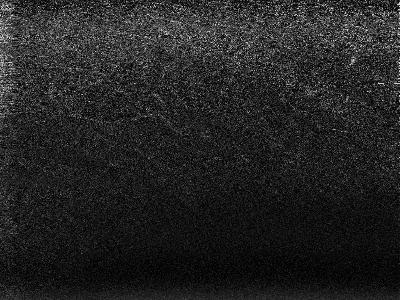

Supplement: Supplementary file 1 — Supplementary Information 1. [file 41598_2024_65534_MOESM1_ESM.gif]

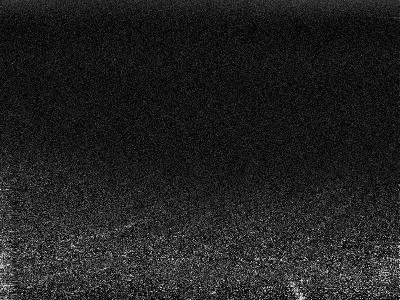

Supplement: Supplementary file 2 — Supplementary Information 2. [file 41598_2024_65534_MOESM2_ESM.gif]

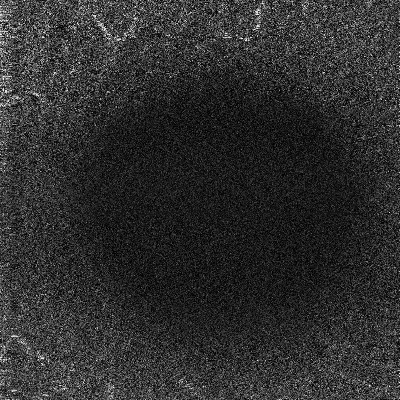

Supplement: Supplementary file 3 — Supplementary Information 3. [file 41598_2024_65534_MOESM3_ESM.gif]

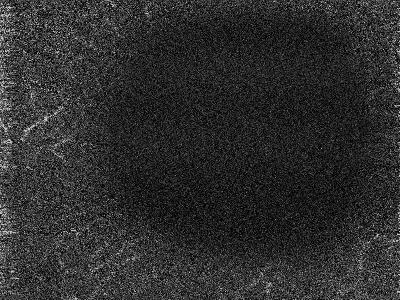

Supplement: Supplementary file 4 — Supplementary Information 4. [file 41598_2024_65534_MOESM4_ESM.gif]

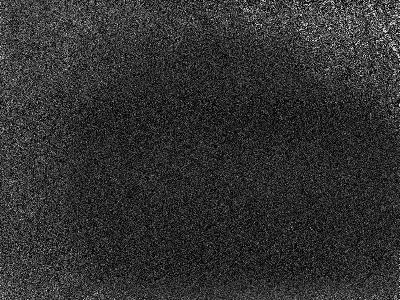

Supplement: Supplementary file 5 — Supplementary Information 5. [file 41598_2024_65534_MOESM5_ESM.gif]
