# Supplementary material for: Spatially resolved imaging of human macular capillaries using adaptive optics-enhanced optical coherence tomography angiography
Source: Sci Rep. 2024 Jul 5;14:15540. doi: 10.1038/s41598-024-65534-y (PMC11226425; doi:10.1038/s41598-024-65534-y)
Supplement: Supplementary file 6 — Supplementary Information 6. [file 41598_2024_65534_MOESM6_ESM.docx]

Supplementary figure 1: Capillary connections between the IVP (top left) and the DVP (bottom right) of subject 1. From A to D, successive *en face* slabs enabling to follow a transverse capillary connecting between the IVP and a venous confluence in the DVP (white arrow).

Supplementary figure 2:

Tracings of perifoveal vessels splitted by capillary layersin subject 1. The diameter of the largest fitting circle in the foveal avascular zone was measured in each layer: in this example, it was 348µm in the SVP, and 598 µm in the IVP and DVP.

Supplementary video 1: AO-OCTA fly-through video of the area shown in figure 2(yellow square area of figure 1).

Supplementary video 2: AO-OCTA fly-through video of the area shown in figure 3, illustrating the venous drainage (blue square area of figure 1).

Supplementary video 3: AO-OCTA fly-through video of the fovea of the right eye of subject 5.

Supplementary video 4: AO-OCTA fly-through video of the fovea of the left eye of subject 9. The PFA is not visible in the SVP but is clearly visible in the IVP and DVP.

Supplementary video 5: AO-OCTA fly-through video of the fovea of the left eye of subject 8 (see also figures 5 and 6).
